# Qualitative Protocol of Chinese Parents and Siblings Experiences of Managing Challenging Behaviours of Adult Persons with Intellectual Disability in Hong Kong and Northern China

**DOI:** 10.3390/ijerph21060673

**Published:** 2024-05-24

**Authors:** Lisa Pau-Le Low, Alice Nga-Lai Kwong, Yue Wang, Maggie Yat-Cheung Wong, Mimi Mei-Ha Tiu, Karen Wing-See Wan

**Affiliations:** 1 School of Health Sciences, Saint Francis University, Tseung Kwan O, Hong Kong 8732, China; nlkwong@sfu.edu.hk (A.N.-L.K.); ycmwong@sfu.edu.hk (M.Y.-C.W.); wswan@sfu.edu.hk (K.W.-S.W.); 2 School of Nursing, Tianjin Medical University, Tianjin 301700, China; yuewangn@163.com; 3 School of Nursing, St. Teresa’s Hospital, Kowloon, Hong Kong 327, China; mimitiu@gmail.com

**Keywords:** caregiving, challenging behaviours, Chinese, intellectual disability, parents, qualitative research, siblings

## Abstract

Background: While the literature has highlighted the immense challenges in caring for family members, it is still unclear what the needs of family carers of persons with intellectual disability and challenging behaviours are and what has worked for them. This study aims to examine 60 parents’ and siblings’ experiences in managing the challenging behaviours of their adult family member with intellectual disability whilst living at home. Methods: A qualitative grounded theory approach using semi-structured interviews will be adopted. Purposive sampling will be used to recruit family carers who live with adult persons with intellectual disability and use one community service in Hong Kong. Three special schools for persons with intellectual disability from northern China will be approached. Results: This study will aim to provide an in-depth understanding of the experiences of family carers and compare the different circumstances they face when managing the challenging behaviours of their adult relatives with intellectual disability in their family home. Conclusions: Although this study targets adults with intellectual disability, the findings will provide a point of reference for adolescents and younger persons who exhibit demanding and challenging behaviours and live with their families. Recommendations can guide the development of appropriate strategies to strengthen services for family carers.

## 1. Introduction

The World Health Organization has highlighted the increasing number of persons with intellectual disability and the need to explore the conditions that would enhance their longevity and general health status [1]. In enabling them to achieve well-being, there is a need to simultaneously recognise the diverse needs of persons with intellectual disability as well as to examine the level of support they can have to help them cope with the changes that can occur across the lifespan. In Hong Kong, the Rehabilitation Programme Plan has set strategic directions and measures to address various service needs, for example, residential and community support services, employment support services, the provision of barrier-free facilities and transport, healthcare, education, sports, and arts [2]. Specific to this project on family caregiving, the consultation report specifically refers to the following factors: (1) enhancing community services to enable persons with disabilities and their families living in the community to obtain necessary support and be admitted to residential care homes when in need, and (2) the ageing of persons with disabilities. Indeed, these consensus initiatives have been set to improve services and support the families of persons with disabilities in the longer term. However, they are rather unspecific and not concerned with the realities of everyday life and having to adjust over the years and accept living with a person with intellectual disability, who may present with specific issues such as behaviours that are considered challenging. In these circumstances, how can family carers overcome the impediments to supporting persons with intellectual disabilities who manifest challenging behaviours to cope and remain at home for as long as possible?

According to the WHO’s latest International Classification of Diseases, intellectual disability is defined as a health condition, known as disorders of intellectual development involving ‘a group of developmental conditions characterised by significant impairment of cognitive functions, which are associated with limitations of learning, adaptive behaviour and skills’ [3]. The literature refers to ‘challenging behaviour’ as including a range of behaviours, such as self-injury, verbal and physical aggression, destructiveness, inappropriate sexualised behaviour and stereotypical behaviours [4,5]. A meta-analysis conducted in 2016 reported a global prevalence ranging from 0.05% to 1.55% [6]. Other literature highlights that approximately 10–20% of people with intellectual disability engage in challenging behaviours [7] that are likely to persist over long periods of time [8]. In mainland China, a national survey found that approximately 75 out of 1000 individuals have been diagnosed with intellectual disability, with a higher prevalence in rural areas (1.02%) compared to urban areas (0.04%) [9]. In Hong Kong, household survey data estimate the prevalence of intellectual disability to be between 1.0 and 1.2% [10].

Scholarly work over the past decade has shed some understanding on the caring issues faced by family carers of persons with intellectual disability. There are more quantitative studies that have been conducted on stressors, perceived stress levels, and coping strategies to manage stress and fewer qualitative studies on carers’ actual experiences. It is learnt that family caregiving is an important concern; loved ones rely on these family carers to bring joy and satisfaction to their lives. Regardless of the severity of intellectual disability, the degree of health and well-being of family members can be affected with time. There is evidence from studies that have counteracted these findings and have shown how family carers have been strengthened and become more supported, confident, and competent in protecting the care recipients, thereby continuing with their caring tasks for much longer [11,12]. A study conducted by the first author [13] generated findings to identify the general and future care support needs for adult persons with mild to moderate intellectual disability and how family carers would be expected to take care of them. Participants were largely parents (usually mothers were the caregivers) as opposed to siblings. The health, medical, and psychological profiles of the carers revealed that, as mothers aged, they experienced degenerating chronic health conditions that needed regular follow-up. They confronted extreme difficulties and became more exhausted with daily activities when attempting to control the emotions and behaviours of their adult children. Being carers meant investing extra attention in observing health changes, providing emotional support, and relentlessly dealing with the behavioural problems of their adult children with intellectual disability. Indeed, the study [13] alluded to the identification of the emotions and behavioural issues of the persons with intellectual disability that warranted attention from the carers, but the management of challenging behaviours was not examined in any depth. In fact, behavioural issues in the family should be promptly, swiftly, and decisively managed before the household becomes a battleground [14].

Although the topic ‘challenging behaviour in the family with intellectual disability’ does not appear to be new, there is no concrete evidence to suggest what will work and help the family. The topic can be potentially sensitive and distressing to talk about, and hesitancy in revealing feelings about their own adult family member with intellectual disability will need careful planning to overcome. A highlight in the family caregiving research on persons with intellectual disability and challenging behaviour reveals the ‘hard work but worth it’ phenomenon [15]. Indeed, studies on challenging behaviours and parents’ attributions, emotions, and behavioural responses have targeted the younger age group [16,17] as opposed to adults with intellectual disability. This suggests that behavioural change and reinforcement may be harder to change at an older age, and the effectiveness of strategies used will need to be monitored. Previous studies show that after individuals with intellectual disability and challenging behaviours have reached adulthood, the caregiving tasks of family carers may have changed, but the amount or intensity of input remains constant [18,19]. Family carers generally experience less support as their family members with intellectual disability leave school and paediatrics-based support and enter adult-based community and medical services [20,21,22]. Furthermore, as family carers grow older, caregiving can be more difficult [8,15]. The literature also points to the need to understand family relationships, dynamics, and harmony with persons with moderate or mild ID, histories of challenging behaviours, and their family members [12]. This refers to an examination of the dynamics, harmony, and processes inherent in sustaining positive family relationships (e.g., reciprocity, flexibility, accommodation, trusting, and expressing affection) versus relationship barriers (e.g., victimizing and behavioural dyscontrol) that can severe family relationships in each household. These notions will be an important underlying basis for understanding how parents and siblings who may live together actually deal with these behaviours while upholding and maintaining family relationships, harmony, and unity in the household.

In the Chinese culture, family is regarded as the most important part of a person’s life, and the emphasis on the filial piety of children in taking care of the psychological well-being of all the family members in the family unit is paramount [23,24]. The emphasis of the Confucius collective view on the practice of filial piety in the family will influence parents’ and siblings’ perspectives. In the Chinese cultural context, this is perhaps more common when adult children show filial piety towards their parents by looking after their older parents in their own home instead of sending them to a nursing home where health professionals provide the care. Caregiver strain experienced by Chinese families affects not only the well-being of caregivers but also the family as a whole, and these families can experience conflictual family relationships, which is reported to be one of the major barriers to accessing support [25]. For carers of persons with intellectual disability in the Chinese context, a glimpse of the general caregiving perceptions and coping styles of Chinese mothers with ID children can be found in Mak and Ho’s early work [26]. The uniqueness and suitability of employing relationship-focused coping strategies were found to fare better in managing stress and burden compared with problem-focused and emotion-focused coping strategies, which are commonly adopted by parents of children with intellectual disability in Western populations [27,28]. For carers rearing children with severe intellectual disability, there is evidence of encountering higher caring stress and impacts on psychological well-being [29]. Surprisingly, there was no mention of ‘managing challenging behaviour’ as a form of stressor. Attention was given to major stressors identified to be related to physical tasks (bathing, toileting, physical handling, and shopping) and concerns for the future. It is noteworthy that the study highlighted that carers adopted internal coping strategies and did not really want to seek help from others, suggesting that it may be best kept in the family [30]. This situation is highlighted in the tragic case of two middle-aged brothers who were starved to death in Hong Kong in 2023 [31]. While their mother was hospitalized, they were left alone, refusing to open their front door and seek help after their food supply ran out. It appeared that the brothers were so dependent on their carer that they became helpless. While the outcome in this case might be considered an outlier, it highlights that caregiving is characterized by the lack of formal support, and cultural concerns such as loss of face, strong affiliated stigma, guilt, and the shame of having an intellectually disabled person in the family [32]. In a society that values self-respect and social standing, Franzen and Feaster [33] refer to the loss of face and stigma that are deeply felt by the participants in a study conducted in Central China, and which contributed to the social exclusion of children with disabilities and led them to be characterized as an ‘invisible’ population.

Comparatively, there is more research on parents than siblings as carers for persons with intellectual disability. However, the international and local literature is relatively sparse and little is known about how family caregivers (parents and siblings) who confront challenging behaviour actually manage it at home. Some solutions have been mentioned by families to manage challenging behaviours at home. These include transition to care homes, uptake of formal community day care services, and using sedation. Family views on the following aspects have been examined: (1) sourcing alternative residential accommodation and making the transition from home to long-term care settings [34]; (2) using the daycare support services to relieve the impact on family life [11], and (3) the use of psychotropic medications to manage challenging behaviours in adults with ID [35,36].

Attention has been given to involving siblings when planning long-term care planning for persons with intellectual disability. There have been reports on concerns about helping siblings to prepare for potential caregiving in the future, especially when ageing parents have passed away [37], and examining the quality of siblings’ lives while living with their siblings with intellectual disability [38,39]. There is a need to understand the values siblings hold in terms of making commitments to being the primary caregiver in the future, and manifesting challenging behaviours would make it harder for them to commit. Indeed, caring for people with intellectual disability and managing their challenging behaviours can cause a substantial burden for Chinese family carers [40].

### Questions to Guide This Study

Some questions that were used to guide this study are as follows:

How do parents and siblings who live together in the same household actually deal with these behaviours while upholding and maintaining family harmony and unity?

Could challenging behaviours in the family be better handled within the home and thereby deter family members from the option of placing the person intellectual disability in a residential care home?

To encourage experience sharing and to off-load the caregiving demands in the family, this study will approach parents and siblings to examine their experiences of managing challenging behaviours in their family home.

## 2. Materials and Methods

### 2.1. Design

Charmaz’s [41] grounded theory design, which focuses on creating conceptual frameworks or theories through building inductive analysis from the data, will be adopted to collect the interview data to examine family members’ experiences of managing the challenging behaviours of adults with intellectual disability living at home. Given that there is a dearth of prior work in this area, this approach is well-suited to this study since it primarily deals with experiences over time and attempts to develop a theory that is grounded in the data. The purpose of the study is to improve theoretical understanding of how family carers manage challenging behaviours of adults with intellectual disability at home and to produce new knowledge that would be accessible to the participants and have the potential to guide actions to inform practice.

### 2.2. Settings and Participants

In Hong Kong, services provided by non-government organizations to persons with intellectual disability, including sheltered workshops, daycare centres, parents’ associations, parents’ resources centres, and churches, will be approached. Residential care services will be excluded. For northern China, family carers of adults (aged over 18 years old) with intellectual disability and challenging behaviours will be recruited via the teachers from three special schools. Adults (aged 18–21 years old) attend the special school, while adults (aged over 21 years old) with intellectual disability can be retrieved from the records of special schools and the Federation of Disabled Persons.

Purposive sampling will be used to recruit family members who can provide rich information and share experiences of the phenomena under study. The inclusion criteria for family members are: (1) aged 18 years and over, either male or female, living at home with persons with intellectual disability, and understands Cantonese (if in Hong Kong) and Mandarin (if in China), (2) immediate family member of persons with a mild to severe grade of intellectual disability and challenging behaviour, (3) parent and sibling who are primary/guardian or secondary/subsidiary caregiver, (4) at least six months of caregiving, and (5) the person with intellectual disability is using a community service or voluntary organisation. If in China, they should be currently attending or have previously attended a special school. The exclusion criteria are family members whose relative with intellectual disability has (1) uncorrected visual or hearing impairments, (2) unstable health conditions such as cancer, and who have had surgery within the last 3 months, (3) ID adults with infections or other active medical conditions that affect their daily lives, or who have a progressive disease (e.g., neuromuscular disease) or a degenerative disorder (e.g., degenerative seizures). It is planned to recruit 60 family members (parents and siblings) from Hong Kong and northern China. Data collection will end when data saturation is reached.

### 2.3. Data Collection

Once the ethics application has been approved, the principal investigator and research staff recruited for the project will visit each site to introduce the study to the administrators or persons in charge to gain access and discuss the process of recruiting eligible family members. Under the supervision of the principal investigator, the research staff will conduct either face-to-face or Zoom interviews according to the preference of family members. An interview guide composed of semi-structured questions has been developed from a review of the literature and will address the objectives of the study. The questions will be validated by a panel of members in the fields of nursing, social work and/or psychology before the main data collection. In Hong Kong and China, a pilot study will be conducted on two family members to test the interview guide and revise the wording if needed. A co-investigator of the project in China will oversee the data collection process and hold regular meetings with the Hong Kong research team. As data will be collected via the teachers at special schools, prior agreement will be sought from the family members before their contact details are passed to the researcher to arrange the interviews. The same approach will also be used to contact the manager of the Federation of Disabled Persons to screen the eligibility of potential participants to join the study. Interviews will be arranged at the family’s home or the different sites. Depending on the participants’ preference, a flexible approach will be adopted to collect the audio-taped interview data by offering either face-to-face individual interviews or online interviews through WeChat or Zoom. Only the audio recording will be kept as data. It is anticipated that interviews will span 45–60 min. Examples of the main topics that will be covered in the interview will include, for example:

Responsibilities for and care given to the family member with intellectual disability.
Family relationship with the person with intellectual disability;
Views and attitudes of challenging behaviours presented by the person with intellectual disability;
Actions and methods taken to deal with the challenging behaviours;
Strategies and support adopted to handle challenging behaviours.

### 2.4. Data Analysis

Concurrent data collection and data analysis will be conducted. All the data collected will be centrally managed in Hong Kong. Once the interview has been completed in China, the files will be password-locked and WeChat to Hong Kong for the research staff to commence data management of the transcripts. Close communication will be held with all research members during the data analysis. Before commencing data analysis, all interview data will be checked for accuracy and will be transcribed verbatim. Each transcript will be checked against the taped recordings to ensure the accuracy of the content and that the translation of terms and/or words is consistently used throughout. Data collected from Hong Kong and China will be managed and analysed separately. Following Charmaz’s method of grounded theory analysis [41], the data will be analysed using the constant comparative analysis methods. Three main phases of coding will be used to analyse the data. First, initial codes will be developed in accordance with the objectives of the study. The process of working through a transcript will involve constantly comparing one part of the participant’s experience with another part by comparing the actions, methods and strategies employed within a transcript to others and between various participants’ experiences. After completing coding on the first ten transcripts, the initial codes will be verified against the data, and a preliminary analytical structure will be formed. Second, focused coding will be conducted. Codes will be re-grouped to broader sub-categories or themes by comparing the data from within and then across the codes/sub-categories to ensure that all incoming data gathered from interviews will be refined and checked. This will generate the concepts that will be used to develop the theoretical model (the theory) through an inductive process of defining, categorising, comparing data, and explaining and seeking relationships in the data [41]. Focused coding will also involve the process of identifying connections between the categories, such as causative factors, contextual circumstances, and action strategies and outcomes. Finally, the project team will work together to fully describe the data and ensure that distinctly independent themes are compiled to develop a ‘comprehensive whole’ to describe the phenomenon under study. Owing to the large amount of interview data in this project, a qualitative data analysis software known as ‘MAXQDA Analytics Pro 2023’ (VERBI Software, Berlin, Germany) will be used to code, organise, and manage the data.

## 3. Discussion

Currently, there is no substantial literature on family care of people with intellectual disability and challenging behaviours. This study will further deepen our understanding of how family carers overcome the impediments to supporting their relative with intellectual disability who manifests challenging behaviours to cope and remain at home as long as possible. Managing challenging behaviour remains one of the most challenging aspects of caregiving for an adult member with an intellectual disability. While the literature has highlighted the immense challenges in caring for family members, the range of needs and what has worked for family carers of persons with intellectual disabilities and challenging behaviours are still unclear. However, prior to being able to pinpoint which initiatives/interventions/programmes will work, there is an utmost need to give full attention to knowing more about what support families actually want to be able to maintain persons with intellectual disability at home as opposed to resorting to residential care homes or other long-term care settings [35,42]. This study will have a significant impact on family carers and persons with intellectual disabilities and will inform public and private expenditures of care. Indeed, there is scope to welcome more new research knowledge in this area.

Detailed descriptive experiences and management strategies employed by parents and siblings when faced with different circumstances while handling the challenging behaviours of persons with intellectual disability in their own homes will be revealed. Findings from this study will provide insights and knowledge into understanding family carers’ strategies for managing challenging behaviours, enable the comparison of data between family carers when faced with different circumstances while handling challenging behaviours, and help us to plan future intervention studies to support family carers in this context. Particularly, it is expected that the findings will provide a better understanding of the characteristics of adults with challenging behaviours and what factors will worsen or improve the behaviours. According to Hastings et al. [43], challenging behaviours serve some important functions for the individual with ID. Understanding the functions that challenging behaviours serve would enable carers to identify whether they can respond in ways likely to solve these problems. Studies like this one are important in being able to hear the experiences and views of parents and siblings who are directly involved in providing day-to-day care. By allowing them to be engaged in the research process from its conception to dissemination, they are the most preferable persons to speak out on the needs of persons with intellectual disability and propose recommendations to instigate changes to service providers and policymakers. Therefore, they are the key stakeholders in making recommendations for developing appropriate and effective strategies to guide the next step of intervention planning to increase the quality of life of both carers and adults with intellectual disability. Although this study targets adults with intellectual disability, reference can be made to transferring the knowledge generated from this study and applying it to adolescents and younger persons who exhibit demanding, challenging behaviours and live with their families.

This study has incorporated adult siblings in the role of family caregiving and the perspectives they hold on contributing to the care of their sibling with intellectual disability. We expect that a longer period of data collection will be needed for recruitment, owing to the unforeseen constraints in recruiting sibling participants from service providers in the community, particularly when parents are usually the point of contact for persons with intellectual disability who use those services. Having a good community network is a bonus for this project, but the research team will need to revisit the recruitment plan as the study progresses. There may be a need to consider snowball sampling and call on participants who have already been interviewed to help increase the pool of potential participants.

## 4. Conclusions

Although this study targets adults with intellectual disability, findings will provide a point of reference for adolescents and younger persons who exhibit demanding and challenging behaviours and live with their families. This study highlights the importance of supporting and educating primary caregivers to have a more scientific way of taking care of adults with ID. The recommendations for developing appropriate and effective strategies to strengthen services for family carers of adults with ID can guide the next step of intervention planning to increase the quality of life of the caregiver and the adult with intellectual disability.

## Data Availability

The data collected from this study are available on request from the corresponding author.
